# Mesoscale Eddies Are Oases for Higher Trophic Marine Life

**DOI:** 10.1371/journal.pone.0030161

**Published:** 2012-01-17

**Authors:** Olav R. Godø, Annette Samuelsen, Gavin J. Macaulay, Ruben Patel, Solfrid Sætre Hjøllo, John Horne, Stein Kaartvedt, Johnny A. Johannessen

**Affiliations:** 1 Institute of Marine Research, Bergen, Norway; 2 Nansen Environmental and Remote Sensing Centre, Bergen, Norway; 3 School of Aquatic and Fishery Sciences, University of Washington, Seattle, Washington, United States of America; 4 Department of Biology, University of Oslo, Oslo, Norway; 5 King Abdullah University of Science and Technology, Thuwal, Saudi Arabia; 6 Geophysical Institute, University of Bergen, Bergen, Norway; Institut Pluridisciplinaire Hubert Curien, France

## Abstract

Mesoscale eddies stimulate biological production in the ocean, but knowledge of energy transfers to higher trophic levels within eddies remains fragmented and not quantified. Increasing the knowledge base is constrained by the inability of traditional sampling methods to adequately sample biological processes at the spatio-temporal scales at which they occur.

By combining satellite and acoustic observations over spatial scales of 10 s of km horizontally and 100 s of m vertically, supported by hydrographical and biological sampling we show that anticyclonic eddies shape distribution and density of marine life from the surface to bathyal depths. Fish feed along density structures of eddies, demonstrating that eddies catalyze energy transfer across trophic levels. Eddies create attractive pelagic habitats, analogous to oases in the desert, for higher trophic level aquatic organisms through enhanced 3-D motion that accumulates and redistributes biomass, contributing to overall bioproduction in the ocean.

Integrating multidisciplinary observation methodologies promoted a new understanding of biophysical interaction in mesoscale eddies. Our findings emphasize the impact of eddies on the patchiness of biomass in the sea and demonstrate that they provide rich feeding habitat for higher trophic marine life.

## Introduction

Eddies advect, mix, and redistribute water masses [1] with significant impacts on the production, distributions, and densities of marine life. Strong associations between the environment and corresponding biological responses are well documented: upwelling stimulates production by renewing nutrient supply to phytoplankton, ultimately leading to increased fish production [2]; turbulence increases the encounter rates between predator and prey and sustains a viable environment for juvenile fish, potentially improving recruitment success [3], [4], [5]. But despite observations of eddies stimulating production at lower trophic levels [6], [7], and biomass accumulation at higher trophic level impacts [8], [9], [10], [11], their importance to oceanic production is poorly quantified. Characterizing physical-biological coupling within eddies is also challenging due to the mismatch of temporal and spatial scales. Eddies develop over time scales of days to weeks [12], while biological responses to changes in the environment can occur within a day (e.g. changes in primary production in response to variation in light or tide [13], [14]). Oceanic eddies [15], [16] spanning tens of kilometres result in patchy, three-dimensional distributions of marine life [17], [18], [19]. Conventional observational methods that combine vessel-deployed instruments [20] and satellite remote sensing data [21] are insufficient to provide synoptic and synchronous three-dimensional views of eddy structure, dynamics, and their biological consequences. This paper demonstrates mapping of anticyclonic mesoscale eddies and quantification of associated distributions of fish and zooplankton by combining data from ship-based platforms (acoustics, mid-water trawls, current profiler, and CTD) with satellite altimetry, and Synthetic Aperture Radar (SAR) data.

## Results

### The discovery

**Figure 1:**
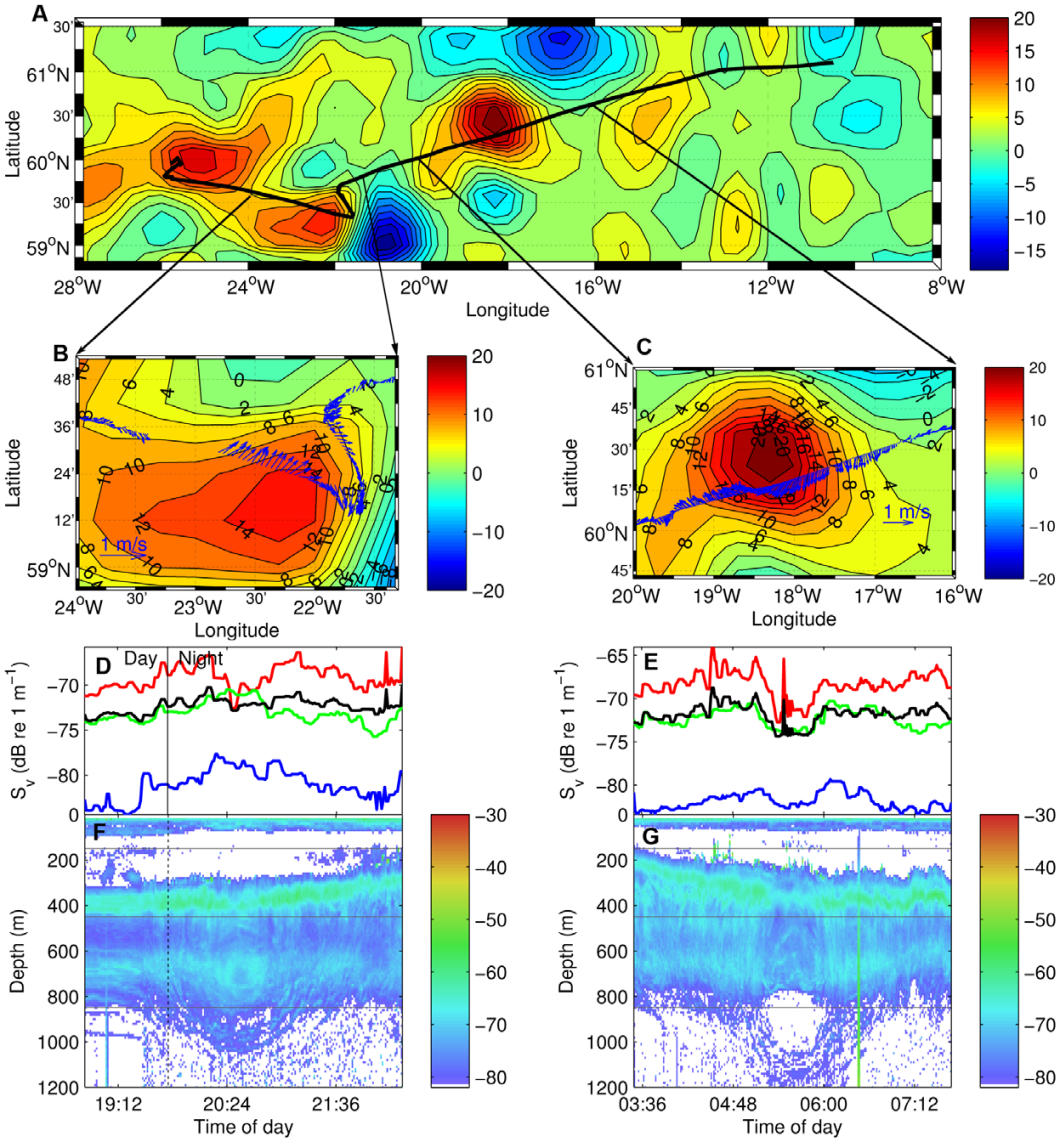
Acoustic, satellite, and ADCP comparisons from a transect through the Iceland Basin eddy. Panel A: ship track (black line) through multiple eddies as detected by satellite altimetry anomalies (colour scale in cm) in June 2004. Panels B and C: co-occurrence between the satellite altimetry anomalies (cm) of two of the eddies and the wheel structured acoustic record (panel F and G, showing S_V_ at 18 kHz, colour scale in dB) of two anticyclonic eddies. Water current velocity vectors (m/s, 0–600 m) along the cruise track are indicated by the blue arrows (panels B and C). Panels D and E illustrate the variation in biomass density (s_V_) in the upper depth layer (150–450 m, red), intermediate depths (451–850 m, green), deep water (>850 m, blue), and entire water column (black). Vertical line in D, F indicate sunset. Sunrise is taking place prior to the start of the horizontal axis in E, G.

During the 2004 Mar-Eco (www.mar-eco.no) expedition to the Mid-Atlantic Ridge [22] we repeatedly observed that oceanic acoustic records (see Material and Methods) in the Iceland Basin delineated a weak but characteristic “wheel shaped” structure extending horizontally 80–100 km and vertically to 1200 m depth (Figure 1F, G). Given their appearance and the close geographical match to anticyclonic (clockwise rotating) eddies detected by satellite altimetry (Figure 1A, B, C, see also Material and Methods), we attributed the observed patterns to the acoustic footprints of biomass structured by eddy dynamics. Four similar acoustic footprints, two of which are shown in Figure 1, were co-located with four anticyclonic eddies. Synchronous ADCP (Acoustic Doppler Current Profiler) measurements showed water flow changing direction when crossing through the centre of the eddy (Figure 1C), further strengthened our inference. Unfortunately, our supposition could not be fully validated due to the lack of oceanographic profiling, and limited biological sampling. The last of four eddies occurred at a predetermined sampling station and was sampled by multiple gears (see Material and Methods).

### The follow up study

**Figure 2:**
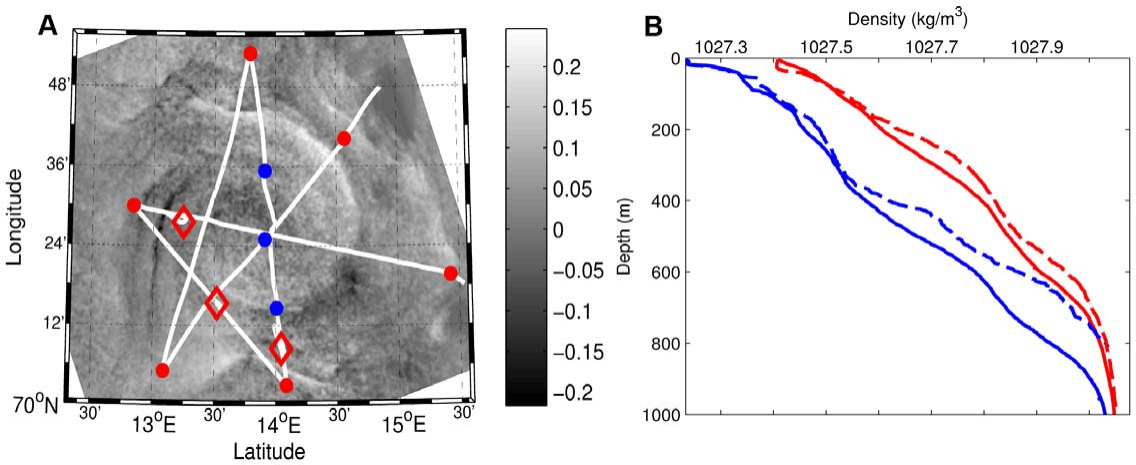
Oceanographic sampling and the origin of the water masses of the Norwegian Sea eddy. Panel A shows cruise tracks with acoustic sampling and CTD casts overlaid on anomalies in the SAR back scatter (dB); blue dots are inside the eddy and red dots along the outer periphery. Red diamonds illustrate net sampling locations. Panel B shows the difference in density (kg/m^3^) of water inside the eddy (solid blue line, average of all blue stations in panel A) and in the outer periphery of the eddy (solid red line, average of all red stations in panel A). Samples from nearby coastal (stippled blue) and offshore waters (stippled red) allow evaluation of the origin of the water masses in the eddy.

**Figure 3:**
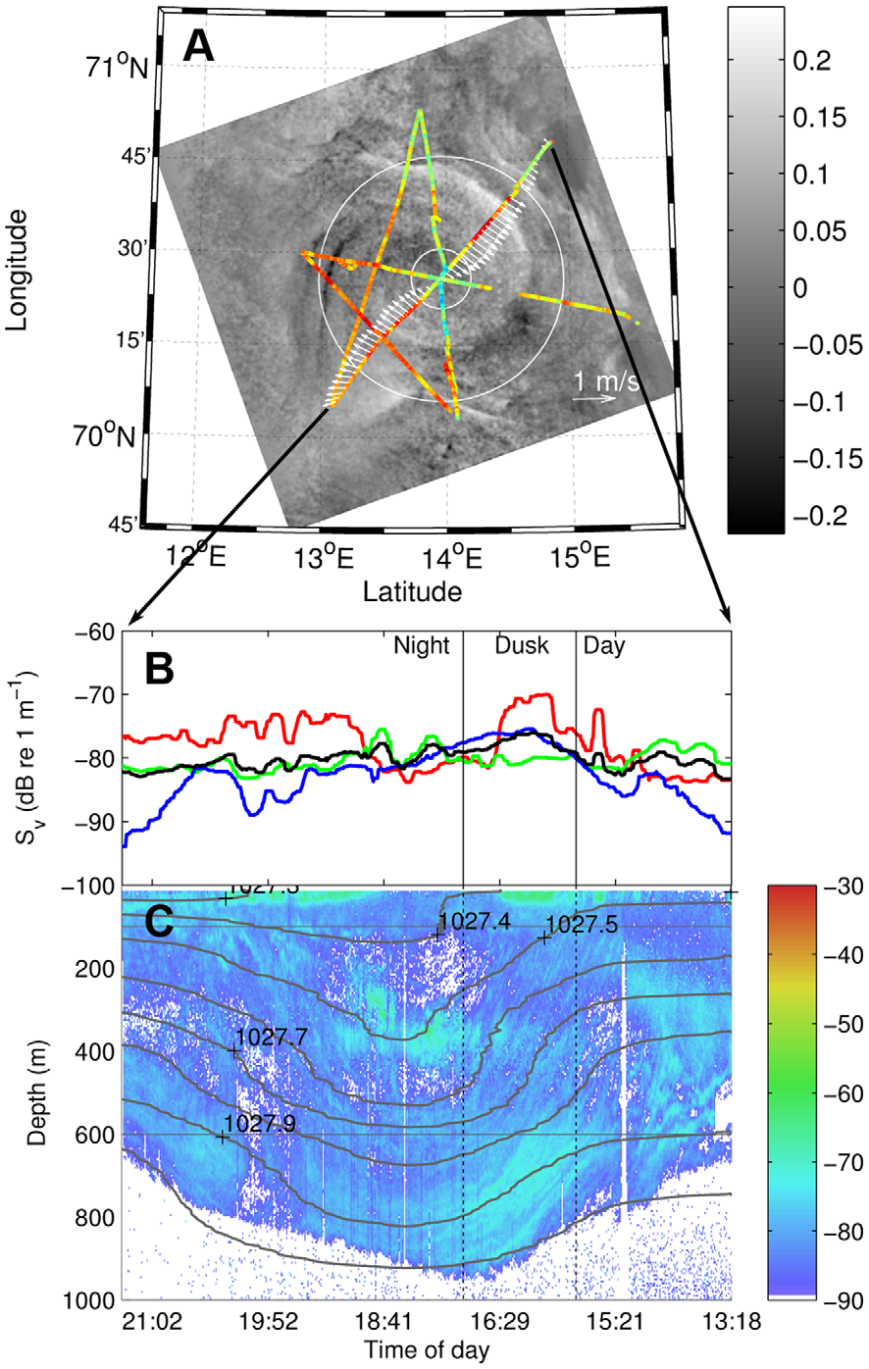
Acoustic, satellite, and CTD data comparisons from a transect through the Norwegian Sea eddy. Panel A shows anomalies in SAR back scatter (dB) overlaid with ADCP current velocity vectors (m/s, 0–600 m) along the cruise track in November 2009. Colours along the track illustrate change in biomass density at surface (accumulated S_V_ over the layer 15–100 m – blue is the lowest observed S_V_ and red is the highest). White circles delineate eddy centre, periphery and outside (see Material and Methods). Panel B shows the variation in average biomass density (S_V_) in the surface scattering layer (15–100 m, red), intermediate depths (101–600 m, green), deep water (601–1000 m, blue), and the entire water column (black). Panel C shows the depth distribution of biomass (S_V_, dB) at 38 kHz over time along the indicated transect through the eddy centre. Water density contours calculated from CTD casts are overlaid on the acoustic data.

To further investigate the observed phenomenon, we designed a study in the Norwegian Sea in November 2009 where oceanographic and acoustic sampling were based on the geographic position and extent of an anticyclonic eddy detected by satellite SAR (see Material and Methods). During two calm days, we sampled the eddy, about 50 km in diameter, using a star pattern (Figure 2A). Ten CTD (Conductivity, Temperature, and Depth) casts (Figure 2A) provided data on water properties within the eddy. Two additional CTD profiles, on and off the neighbouring shelf, were used for comparative purposes (Figure 2B). Three mid-water trawls identified biological constituents in the acoustic record (Figure 2A). ADCP measurements provided continuous current velocity profiles along the ship track (Figure 3A) and continuous vertical profiles of biomass density were obtained from the echosounder (Figure 3C).

Data from CTD profiles and the ship mounted ADCP were used to characterise physical properties of the Norwegian Sea eddy (Figures 2, 3). The core water of the anticyclonic eddy was warmer, less saline, and less dense, than water in the eddy periphery (Figure 2B). Similarities with samples from nearby coastal (Figure 2B, stippled blue) and offshore (Figure 2B, stippled red) stations suggest that the eddy originated from inshore waters and migrated into deeper offshore water which encircled the coastal water core at the time of our observations. This pattern has been observed in other mesoscale eddies [23], [24], [25], and specifically in the Gulf of Alaska [26] with subsequent impacts on larval fish distribution. As expected, ADCP measurements showed that water flow changed direction across the eddy centre (Figure 3A), with a persistent direction from the surface to 600 m. The bowl shape of the density structure (Figure 3C) along a section from northeast to southwest conforms to that expected for an anticyclonic eddy [6], [7]. The acoustically-detected horizontal and vertical biomass density along the same transect changed across the eddy, reflecting the same bowl shape as the density structure (Figure 3C). One notable difference is that the vertical biomass distribution extends to the surface, resulting in a bowl shape in the acoustic data rather than the wheel shape observed in the Iceland Basin. The satellite SAR image shows that the eddy is horizontally asymmetric (the strength and character of the SAR signal change from west to east as noted in Figure 3A). This is also reflected in the surface density structure and vertical acoustic records (Figure 3A, C).

### Comparison of biomass structure of anticyclonic eddies

Biomass distribution patterns contained interesting similarities and differences in the two areas. In both cases the dominant pattern in the backscatter switched from horizontal to vertical when going from the outside to the centre of the eddy. The characteristic bowl shape, co-occurring with density isolines, started when the acoustic record of the deep scattering layer (DSL) [27] shifted towards a vertical orientation in the periphery of the eddy, continuing to the bottom of the bowl at approximately 1000 m depth (Figure 3C). The DSL in the Iceland Basin eddies had the same bowl shape (Figures 1F, G) but extended a little deeper (to 1200 m). In the Norwegian Sea the vertically oriented structures intersected the ocean surface while the vertical stratification of the upper part of the DSL in the Iceland Basin curved to form a dome shape at about 200 m, giving the acoustic footprint of a submerged “wheel.” Oceanographic observations are lacking in the Iceland basin, but assuming an analogous match between the shapes of the acoustic records and the thermo-haline properties in eddies at the two locations, we infer that the Iceland Basin eddies were mode water eddies [6], [7].

In both study areas we observed slightly higher biomass densities (S_V_) above the steepest change in orientation of the acoustic record (compare Figures 1D and 1F, 1E and 1G, and 3B and 3C). This change co-occurred with the steepest density isolines (Figure 3C). Minimum upper layer densities (Figure 1D, 1E, and 3B, red lines) occurred at the eddy centre, and a maximum in the eddy periphery, which generally contained the highest acoustically measured biomass. Similar patterns were observed in the two deeper layers of the first eddy in the Iceland basin (Figure 1E), while the biomass in the deeper layers of the second eddy peaked at the eddy centre (Figure 1D). In general it appears that acoustically-detected biomass was patchy, corresponding to the structure of thermo-haline isolines. If so, a survey transect that passed through the eddy, but not through the centre will miss the centre minimum, as seen in the second eddy in the Iceland basin (Figure 1D, F). The centre biomass minimum seems to be another common feature for eddies in both areas. In the Iceland Basin the minimum was associated with the centre of the wheel, while in the Norwegian Sea it was clearest in the upper part of the water column.

### Biological sampling

Midwater trawl catches from both areas showed that fish with gas-filled swimbladders dominated the acoustic records. Adult blue whiting (*Micromesistius poutassou*) 27–33 cm length, lantern fishes (*Benthosema* sp.) 2–7 cm, pearlside (*Maurolicus muellerii*) 3–7 cm, and krill (*Meganyctiphanes norvegica*) dominated catches of two surface trawls and one at 280–330 m that targeted the periphery of the Norwegian Sea eddy (Table 1). Pearlside was not a major constituent in the deepest tow. Lantern fish and pearlside perform daily vertical migrations that can span 100 s of meters [28] [27], while blue whiting, the largest and fastest swimmer, normally occupy deep water (300–500 m) with limited vertical movements [29]. In the Iceland Basin we evaluated the species composition at a station in the last eddy. Catches from a pelagic trawl with a multiple opening-closing net and a vertical profiling zooplankton net showed that the dominant organisms contributing to the acoustic backscatter were swimbladdered, mesopelagic fish ranging in length from 3 to 68 cm (Table 2).

**Table 1:** Number and weight of fish species caught at three trawl stations in the Norwegian Sea.

Tow number/Depth	#1/280–330 m		#2/surface		#3/surface		
Time	08 UTC		18 UTC		23 UTC		
	n	w (kg)	n	w (kg)	n	w (kg)	length (mm)
Blue whiting (*Micromesistius poutassou*)	45	7.7	8	1.1	8	1.1	270–330
Lantern fish (*Bentosema* sp.)	466	0.9			289	0.3	27–70
Pearlsides (*Maurolicus muelleri*)	394	0.5					38–65
Northern krill (*Meganyctiphanes norvegica*)		0.2		0.1		0.1	
Herring (*Clupea harengus*)			2	0.6			335–345
Lumpsucker (*Cyclopterus lumpus)*	1				1		
Redfish (*Sebastes* sp.)	1						

Catch (numbers, n and weight, w) composition from the Norwegian Sea eddy interior (station positions are indicated in Figure 2) as a result of targeted trawling on high acoustic densities in the DSL (#1) and on surface concentration (#2 and 3).

**Table 2:** Catch composition by depth strata from the Iceland Basin trawl samples.

		Depth range					
Group	Length Range	0–∼200 m		∼200–800 m		800–1500 m	
	(mm)	FT	KT	FT	KT	FT	KT
Fish-GSB	26–680	31.3	989.4	951.6	196.1	–	199.6
Fish-RSB	135–169	0.0	5.1	46.2	15.1	–	38.3
Fish-NSB	70–251	4.1	80.6	223.7	21.9	–	33.6
Squid	19–288	1.0	218.4	22.8	0.0	–	32.0
Jellyfish		8.7	26.0	946.0	325.0	–	0.0
Macro-crustaceans		0.1	43.8	131.8	46.7	–	34.8
Copepods (no. m^−3^)	–	247.3		99.3		7.2	

The major groups of species represented in the samples organised according to their acoustic properties. Fish with gas-filled swimbladders (Fish-GSB) give echoes of an order of magnitude higher than fish with regressed swimbladders (Fish-RSB, swimbladders regress with age and becomes lipid filled), which give a much stronger signal than fish without swimbladders (Fish-NSB) of same size. Squid, jellyfish and crustaceans are relatively weak acoustic reflectors compared to fish. Catch data (kg) are from pelagic fish trawls (FT) and krill trawls (KT, g km^−1^), except copepods caught by a multinet (no. m^−3^) taken at the end of the vessel track in Figure 1. Copepods contribute little to the recorded acoustic signal. Lengths are standard length for fish and mantle length for squid. Actual fish trawl depth ranges were: FT = 150–200 m, 370–750 m; KT = 10–175 m, 180–845 m, 880–1545 m.

### Temporal dynamics

**Figure 4:**
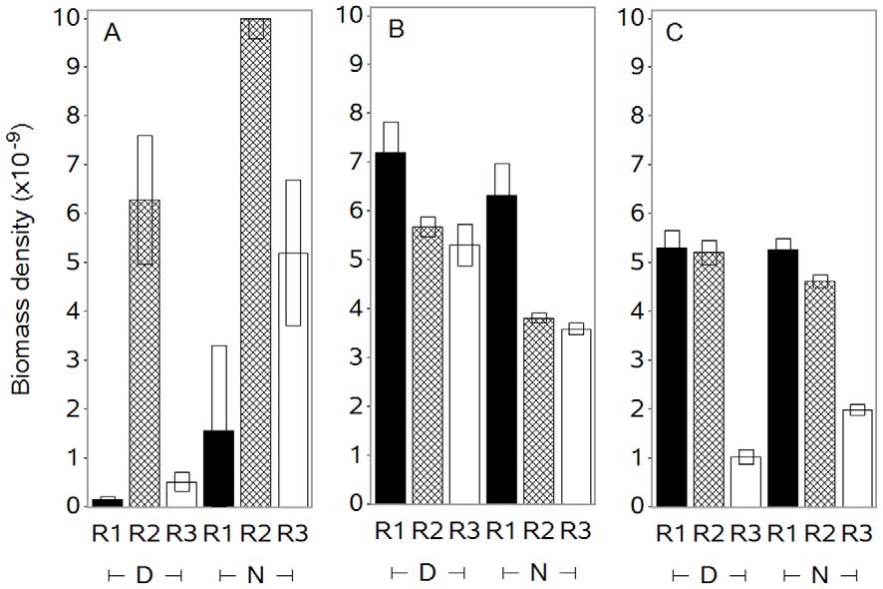
Comparison of acoustic biomass densities (s_V_) related to distance from eddy centre and depth. The three panels show data for the three depth categories; A. surface layer (0–100 m), B. mid water (101–600 m) and C. deep water (Deep, ≥600 m). In each panel distance from centre are categorized in R1<9 km, 9≤R2<37 km, R3≥37 and day (D) and night (N) data are presented.

Marine organisms, like those identified here, can undertake extensive diel vertical migrations. It is possible that the observed patterns could be coincidental and due to the timing of the sampling. In the Iceland Basin, data from a full diurnal cycle was not available for any of the eddies. The diel signal is apparent as a downward migration in the acoustic mid water layer (100–400 m) during early morning (Figure 1G), and an upward migration of the same layer at night (Figure 1F). This movement was not coordinated with the “wheel shaped” signal in the eddy structure: the maximum sun elevation occurred at 11 UTC, outside the time frame of the eddy observations. For the northeast to southwest section in the Norwegian Sea (Figure 3B), the maximum backscatter depth occurred at dusk/night and not at midday as would be expected if animals were undergoing upward migration at night. The combination of all data, organised in a radial coordinate system (Material and Methods), covers three diurnal cycles. These data (Figure 4) support the hypothesis that the acoustic density structures are caused by the eddy (Figure 3C) as follows: the biomass density at the surface is very low close to the centre (Figure 4A, R1), peaking towards the periphery (R2) and again reducing in the eddy outskirts (R3). At medium and deep waters the maxima occurs in the centre and gradually reduces towards the periphery, in most cases with non-overlapping confidence limits. Also, Figure 4 shows a diel effect where densities increase at night at the surface and decrease in mid water. Our interpretation is that distribution and vertical migration occur along the structure of the eddy, thus maintaining the wheel or bowl appearance of the acoustic record. We ran a General Linear Model (GLM, see Material and Methods) with S_V_ as response variable and day/night (t), radius (r) and depth (D) categories (same as in Figure 4) as explanatory variables. Exploring effects and interaction effects showed that distance from centre (r) and depth (D) are the most influential variables while impact of time is not significant. The model including the three category variables and interaction among them explains 78% of the variation (Table 3). These analyses support our earlier interpretation from Figure 4, that biomass of higher trophic marine life is distributed along the eddy periphery, and the distribution pattern is slightly modified by the diel vertical migration.

**Table 3:** Results from the final GLM run (r^2^ = 0.78).

Source	DF	Type III SS	Mean Square	F Value	Pr>F
**D**	2	342.40	171.20	10.30	<0.01
**R**	2	128.16	64.08	3.86	0.03
**T**	1	28.05	28.05	1.69	0.20
D*r*t	12	566.70	47.23	2.84	0.01

## Discussion

### How our study differs from others

Earlier studies have demonstrated structural similarities between physical properties of the eddy and the distribution of lower trophic level biological production [6], [30], [31], [32]. We have demonstrated parallel similarities for higher trophic level marine life. Concentrations of higher trophic marine life, including fish, have been associated with eddies [11], [33], [34], [35], but the low resolution of these observations does not allow comparisons of distribution patterns with eddy structure. We have investigated biological density distribution in eddies by using satellite images to direct acoustic data acquisition along our cruise track to collect continuous data on biomass. Integrated sampling by echosounders, ADCP, and a minimum of midwater trawls, and CTD casts supported quick 3D mapping of the eddy structures with limited temporal lag and spatiotemporal confounding of data streams. This approach provides a unique opportunity to examine connections between physical properties of eddies and their biological responses at high spatial and temporal resolution. This integrated approach contrasts earlier studies where spatial and temporal resolutions of biological data are limited by the sampling methodologies, such as vertical and horizontal net tows.

### Which organisms do we see with acoustics?

In both study areas the acoustic signature of mesoscale eddies originated from swimbladdered fish within the DSL. In the Norwegian Sea blue whiting dominated all of the catches. This is a physioclist fish with a high target strength compared to the other fish species (e.g. lantern fish and pearlside) caught in midwater trawls and is expected to dominate the acoustic records at 38 kHz. Blue whiting is expected to be undersampled in trawl catches due to strong trawl-vessel avoidance [36]. In contrast, swimbladder resonance will increase acoustic reverberation of small mesopelagic fish at the acoustic frequencies used during the surveys [37], [38]. Krill were observed in the Norwegian Sea trawl samples but are also undersampled due to selectivity of the net. Krill will contribute little to the backscatter at 38 kHz but will be seen at higher frequencies [39]. Thus, a partitioning of acoustic contributions from the species observed during the surveys would require additional sampling, backscatter modelling [40], and behaviour studies to determine depth distributions and orientations. As such, the use of acoustic backscatter measures (S_V_, s_V_) to quantify biomass densities over depth and time ranges is not strictly correct for two reasons. First, biomass estimates depend on the acoustic properties and species mix of the ensonified animals, which can change with depth and spatial location, thus making biomass densities not entirely comparable over time and space. Secondly, vertical migration can change swimbladder volume and animal orientations, thus affecting the relationship between acoustic measures and biomass density [38]. For the outcome of this paper these potential sources of uncertainty are considered negligible because biomass structures rather than exact species compositions are the focus.

### Mechanisms behind the biological – physical interaction

In both study areas we infer that acoustic signatures primarily originate from eddy manipulated distributions of organisms within the DSL. Lower trophic level marine production through ‘eddy-pumping’ is well documented [6], [7], [41]. In this study we demonstrate that the restructuring and concentration of biomass caused by eddy dynamics create a rich habitat that can lead to enhanced higher trophic transfer compared to the surrounding waters. The observed result is probably a combination of active and passive biological responses to physical forcing. Even small copepods can be concentrated through active navigation in currents, which optimizes their position relative to suitable prey in productive ocean structures [42]. Similar behaviour by fish feeding on plankton will amplify observed patterns of acoustically-detectable biomass. In addition to extensive diel migrations as seen in the upper part of the water column, lantern fish also exhibit a lethargic behaviour [28] where they can be passively displaced by changes in water mass density or currents [43]. The combination of swimming and passive transport caused by eddy dynamics, enables mesopelagic fish to track concentrations of zooplankton prey. Lethargic mesopelagic fish in deep water may float along water masses of equal density, resulting in the lower bowl shaped portion of the “wheel” in the Iceland Basin [28]. The upper dome of the wheel is located in the epi- or meso-pelagic depth layer, which is dominated by vertically migrating organisms. The enhanced biomass along the shallow dome of the “wheel” will attract mobile animals, even small plankton [42]. The concentrated biomass in this area forms a prey-rich depth zone for vertically migrating fish, foraging along eddy isopycnals as a part of their diel vertical migration. Similarly, a passive or semi-passive concentration of lower trophic level biomass along the isopycnals may attract large shoaling fish. The preservation of the horizontal structure during diel migration in the Norwegian Sea (Figure 4) further suggests that migration takes place in accordance with eddy structure. Blue whiting is probably not part of this migration. Stomachs of fish caught from trawls in surface waters were full of krill and had fully inflated intact swimbladders, indicating adaptation to surface pressures. This contrasts with blue whiting caught in deep water, which typically have ruptured swimbladders when brought to the surface. The surface-caught blue whiting appeared to use eddies as enhanced feeding stations (oases). The occurrence of blue whiting at the surface is considered a response to eddy conditions. All these taxa are deep water species, and must have been entrained in or attracted to the eddy after it left the continental shelf. This hypothesized movement potentially explains their reduced densities at the core of the eddy, which is composed of coastal water. Fish association with eddies has been inferred on the basis of tagging studies of the slow-moving sun fish (*Mola mola*) [33] and feeding concentrations of highly migratory fish such as albacore (*Thunnus alalunga*), blue fin tuna (*Thunnus thunnus)* [34], and even demersal fish species [35]. Analysis within these studies is limited to a geographical comparison of eddy and fish distributions.

The bioproduction of eddies is enhanced by nutrient pumping from deep water to the euphotic zone [6], [7], [41]. Given that biomass accumulation was observed in both winter (Norwegian Sea) and summer (Iceland Basin), we propose that the phenomenon discussed here is mainly driven by an eddy's ability to accumulate and concentrate biomass within the eddy, thereby creating a valuable habitat (oasis) for mobile predators. Primary production at high latitudes is insignificant in November due to the lack of solar radiation. Nutrient pumping to the photic zone [41], has a minor impact on the primary production in the Norwegian Sea at this time. In the Iceland Basin in June primary production could be a contributing factor to enhanced biomass concentration. Yet, enhanced biological production involving transfer of biomass across trophic levels occurs on time scales that extends beyond the lifetime of individual eddies. We consequently attribute the high fish concentrations within the eddy to predators actively searching for food in concentrated prey habitats. For example, the limited vision range of fish larvae requires prey concentrations above certain density levels to enable feeding success [44], and such high concentrations occur often under special physical conditions, e.g. in thin layers [45]. Our findings also suggest that mesoscale eddies provide an optimal set of conditions for enriched feeding for higher trophic marine life in the open ocean, including fish larvae, that would not exist outside of a mesoscale eddy. The biomass minimum found in the centre of eddies suggests that these locations are uninteresting feeding habitats for fish. The origin and development of such minima warrants additional research.

Understanding the impact of eddy dynamics on biomass at different trophic levels is challenging because of the temporal mismatch of eddy formation and decay [16] relative to the transfer of energy from phytoplankton to adult fish. The time required to map mesoscale eddies using vessel based ADCP profiles and CTD casts may also mask observation of coupled biological-physical responses within entrained water masses [14]. Our approach resolves some of these technical challenges, demonstrates that eddies attract higher trophic level organisms, and that biological energy cascades up through the food web even during seasons of low productivity. Quantifying biological responses to water dynamics has been difficult due to the lack of coincident and integrated observations of biological-physical coupling. Our observations at two different locations in two different seasons signify the general applicability of our approach for studying the biological impact of eddies on higher trophic marine life. The use of acoustics to detect and quantify physical and biophysical phenomena has been repeatedly demonstrated [46], [47], [48]. This study demonstrates that an expanded approach combining satellite, net catches, ADCP profiles, CTD casts and acoustic observations provides a new approach to understand and quantify biophysical interactions. Using satellite information to direct the hydrodynamic and acoustic sampling and then acoustic sampling to target collection of biological samples has proved an efficient way of collecting quantitative information about physical-biological interactions.

### Future development

Our definition of inside and outside an eddy is a subjective evaluation derived from satellite and acoustic data. Both study areas are dominated by mesoscale activities (e.g. Figure 1A) and it may be difficult to find unaffected background densities of higher trophic marine life. Also, there were clear indications of asymmetric biomass distributions within eddies, which were not considered in our analysis. The explanatory power of the GLM model would probably be higher without distributional differentiation among the six legs used in the analysis. The continuous high resolution of acoustic technologies enables us to incorporate these observations in future sampling efforts. Future studies should delineate the geographic and physical-biological impact volume of eddies through acoustically informed stratification of oceanographic and biological sampling. Further, studies of density distribution of higher trophic marine life in cyclonic eddies and eddies of different age are interesting challenges where our remote sensing approach could contribute new scientific knowledge. We believe that execution of similar multidisciplinary data collection will create new insights into the patchiness of biological production and biomass distribution in the ocean, including commercially important harvestable biomass and larval fish survival when entrained in rich habitats such as eddies [49].

## Materials and Methods

\The acoustic instrumentation included a Simrad EK60 multi-frequency echosounder system transmitting at 7-second intervals and produced estimates of acoustic volume backscattering strength (S_V_) [50], a logarithmic variable related to biomass density. S_V_ at 18 and 38 kHz was used to visualize physical-biological coupling within eddy structures through the entire water column. To statistically compare biological densities across depth and time strata we used the linear variable volume backscattering coefficient s_V_ which is related to S_V_ through the equation S_V_ = 10 log10 (s_v_) [50]. The calibrated echosounder was operated under high signal to noise conditions from the R/V G.O. Sars [36] which permitted the use of low acoustic thresholds (−85 to −95 dB re 1 m^−1^) to extend the detected organism size range. A 75 kHz ADCP transmitted between echosounder transmissions to measure water velocities, averaged in 50 m depth bins from the surface to 600 m. Data were collected on June 7–9 2004 and on November 16–19 2009 at a speed of 11 knots. The 2004 data were collected when transiting (Figure 1) towards the first station of the Mar-Eco expedition [51], [52]. To sample biological constituents we used fish and macro zooplankton trawls with 3 and 5 independent small-meshed codends (22 mm and 6 mm respectively), for depth-stratified sampling, and a multinet system with eight codends for vertical plankton sampling [53]. The same fish trawl with a single codend was used in 2009.

Sea-level anomaly (SLA) maps from 2004 were merged from satellite altimeter tracks taken during one week around the period of acoustic observations. In 2009 we used Synthetic Aperture Radar (SAR) information recorded one day before the acoustic survey to locate eddies. The satellite data products are available from Collecte Localisation Satellites (CLS), France.

Hydrographic measurements including temperature and salinity were recorded from hull mounted sensors and CTD casts. Estimates of the water density along each transect (Figure 3C) were obtained by cubic interpolation of the density calculated from the CTD casts.

Acoustic data are spatially correlated and thus not independent measurements. In the statistical comparison of the 2009 acoustic density data we therefore averaged the observed acoustic backscatter (s_V_) over three radial categories; centre (R1, <9 km), periphery (R2, 9–37 km), outside (R3, ≥37 km). We also categorized according to depth stratum; surface layer (Shal, 0–100 m), mid water (Med, 101–600 m) and deep water (Deep, ≥600 m). The sun was below the horizon during the cruise, but we observed time-dependent behaviour patterns within the acoustic record. The data were split by day, night and twilight according to the nautical day/night definition. Nautical twilight is the period when the centre of the sun is between 6 and 12 degrees below horizon. Day and night are when the sun is above and below twilight elevations. Finally, the six legs spanning from the centre to the periphery (Figure 1) are assumed to be independent replicates. We studied possible impacts on the observed biomass density (b) by the category variables depth stratum (D), radius (r) and day/night (t), as defined above, through the following generalised linear model (GLM)
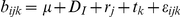
where μ is the overall mean term, D*_i_*, r*_j_*, t*_k_*, the terms relative to the effect of the ith depth category, jth radius and kth time period, and ε*_ijk_* is the error term. Impact of main factors and various interactions were explored. To reduce impacts from extreme values the GLM used the logarithmic S_V_ as a proxy for biomass density (b).
